# Large-Scale Diversity of Slope Fishes: Pattern Inconsistency between Multiple Diversity Indices

**DOI:** 10.1371/journal.pone.0066753

**Published:** 2013-07-03

**Authors:** Jean-Claude Gaertner, Porza Maiorano, Bastien Mérigot, Francesco Colloca, Chrissi-Yianna Politou, Luis Gil De Sola, Jacques A. Bertrand, Matteo Murenu, Jean-Pierre Durbec, Argyris Kallianiotis, Alessandro Mannini

**Affiliations:** 1 UMR-241 EIO (UPF, IRD, Ifremer, ILM), Université de Polynésie française, BP 6570 - Faa’a, Tahiti, Polynésie française; 2 UMR-241 EIO (UPF, IRD, Ifremer, ILM), IRD, Centre d’Arue, Papeete, Tahiti, Polynésie française; 3 Department of Zoology, University of Bari, Bari, Italy; 4 Université de Montpellier 2, UMR 212 Ecosystèmes Marins Exploités (EME), Centre de Recherche halieutique Méditerranéenne et Tropicale, Sète, France; 5 Department of Animal and Human Biology, University of Rome ‘La Sapienza’, Rome, Italy; 6 Hellenic Center for Marine Research, Institute of Marine Biological Resources, Helliniko, Greece; 7 Centro Oceanográphico de Málaga (IEO), Fuengirola (Malaga), Spain; 8 IFREMER, Département Ecologie et modèles pour l’halieutique, Nantes, France; 9 Università degli Studi di Cagliari, Dipartimento Biologia Animale ed Ecologia, Cagliari, Italy; 10 Centre d’Océanologie de Marseille, UMR-MIO, Université d’Aix-Marseille, Marseille, France; 11 National Agricultural Research Foundation, Fisheries Research Institute, Kavala, Greece; 12 University of Genova – Dip.Te.Ris. –Genova, Italy; Technical University of Denmark, Denmark

## Abstract

Large-scale studies focused on the diversity of continental slope ecosystems are still rare, usually restricted to a limited number of diversity indices and mainly based on the empirical comparison of heterogeneous local data sets. In contrast, we investigate large-scale fish diversity on the basis of multiple diversity indices and using 1454 standardized trawl hauls collected throughout the upper and middle slope of the whole northern Mediterranean Sea (36°3′- 45°7′ N; 5°3′W - 28°E). We have analyzed (1) the empirical relationships between a set of 11 diversity indices in order to assess their degree of complementarity/redundancy and (2) the consistency of spatial patterns exhibited by each of the complementary groups of indices. Regarding species richness, our results contrasted both the traditional view based on the hump-shaped theory for bathymetric pattern and the commonly-admitted hypothesis of a large-scale decreasing trend correlated with a similar gradient of primary production in the Mediterranean Sea. More generally, we found that the components of slope fish diversity we analyzed did not always show a consistent pattern of distribution according either to depth or to spatial areas, suggesting that they are not driven by the same factors. These results, which stress the need to extend the number of indices traditionally considered in diversity monitoring networks, could provide a basis for rethinking not only the methodological approach used in monitoring systems, but also the definition of priority zones for protection. Finally, our results call into question the feasibility of properly investigating large-scale diversity patterns using a widespread approach in ecology, which is based on the compilation of pre-existing heterogeneous and disparate data sets, in particular when focusing on indices that are very sensitive to sampling design standardization, such as species richness.

## Introduction

The monitoring of diversity patterns and the identification of structuring factors through large-scale analyses are increasingly in demand [1], [2]. However, to date, most attempts at analysing large-scale diversity patterns have suffered from two major limitations. Firstly, in numerous ecosystems, due to the difficulties and costs of undertaking field studies in order to collect standardized diversity data, large scale studies have been mainly based on the empirical compilation and comparison of disparate pre-existing local data sets, collected for different purposes using different sampling designs. This very widespread approach is the best available in situations where standardized sampling programmes are lacking at the scale of the whole study zone (as is often the case when increasing the spatial scales of investigation). However, the contribution of these studies is usually restricted to rough inter-area comparisons, mostly conducted at coarse grain-scale resolution. Secondly, most of the large-scale diversity studies have only dealt with a limited number of diversity indices, such as species richness and/or heterogeneous indices (*e.g.* the Shannon index), while several studies, have highlighted the limitations of such analyses [3], [4]. In contrast, investigating diversity through multiple indices could be a fruitful alternative approach [5], [6], [7]. It could in particular be used, as a preliminary step towards assessing whether the main diversity components (species richness, evenness, taxonomic diversity, etc.) of a given taxon are driven by the same factors, and respond in a single manner (same direction, same strength, etc.) to a given structuring factor. Despite this, studies that simultaneously analyse multiple diversity indices on the basis of standardized data sets are still few and far between and – when they exist – are mainly restricted to small spatial scales.

This general situation is particularly apparent with regard to marine benthic communities living in the deep ecosystems [8], even for both commercially and ecologically important taxa, such as fishes. For instance, although the continental slope is expected to play a “crucial role in the functioning of the global ecosystem” [9], slope fish diversity has been far less widely investigated than that of coastal ecosystems. In addition, most of the studies measuring the spatial distribution of slope fish diversity worldwide have been carried out at small scales (e.g. [10], [11]) and very few large-scale studies have been based on standardized sampling programmes. The rare large-scale studies of this kind have been restricted to a very limited number of indices, most often only related to estimates of the number of species (e.g. [12]). In this general context, the variation in species richness along the bathymetric gradient has been one of the most widely adopted focal points for investigating slope fish diversity [13], [14]. According to the most popular theory, bathymetric patterns of species richness are expected to be described by hump-shaped curves, so that peak diversity occurs at some intermediate level [15], [16], [17], [18]. Another major issue related to groundfish diversity, common for both shallow and deep ecosystems, is the expected relationship between species richness and primary production or productivity (see [14]). The related paradigm, notably issued from the energy-richness hypothesis [19] **,** predicts a positive relationship between these two descriptors (see [20], [21], [22] among others**)**.

We here propose the first analysis in the deep sea ecosystem to investigate the consistency of spatial patterns of fish diversity assemblages exhibited by several complementary diversity indices according to either depth or spatial area, notably contrasted in primary production levels. Based on the analysis of a set of standardized data, our study is focused on the upper and middle parts of the continental slope of the whole northern Mediterranean Sea.

## Materials and Methods

### Study Area

The Mediterranean Sea has been considered as a “miniature ocean” that can be used as a “giant mesocosm” for better understanding and anticipating the response of the global oceans to various kinds of disturbances [23]. This sea - which has been recognized as a priority area for conservation for several decades (Barcelona Convention, 1976) - has been exposed to strong impact from surrounding catchment basins and the consequences of global climatic changes, notably in the deep ecosystem [9], [24], [25]. While the Mediterranean Sea has been considered as a model for investigating the distribution of deep-sea biodiversity along longitudinal and bathymetric gradients across different areas [26], knowledge on benthic diversity patterns at large scale is still scarce. For most of the taxa, including those living deeper than the continental shelf, the authors of most studies agree with the hypothesis of the existence of a large-scale decreasing trend in species richness correlated with a similar gradient of primary production (see for instance [27], [28], and [29]). However, this paradigm is mainly (if not totally) based on the comparison of disparate pre-existing data sets, sometimes combined with expert opinions (see [29]), and usually restricted to coarse spatial grain resolution.

With regard to fishes, most of the studies focused on - or at least partly dedicated to - the slope ecosystem on the basis of standardized data have been carried out at small regional scales [10], [31], [32], [33], [34], and very few of them were based on the analysis of multiple complementary indices [33], [35], [36]. To our knowledge, the only study providing information on slope fish diversity at large scale on a standardized basis was restricted to the comparison of three small dispersed areas [37] and limited to two of the most traditional diversity indices (i.e. species richness and Shannon index).

### Experiments and Sampling Design

**Figure 1:**
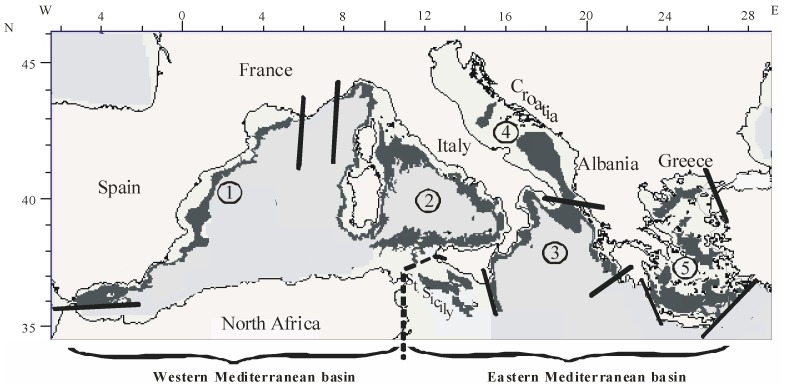
Map of the area studied with boundaries of the spatial units. The sampling zone (200–800 m) is shown in dark grey. The spatial boundaries of the biogeographical zones are delimited by thick black lines. Codes of biogeographical zones: Iberian-Lions, Tyrhhenian, Ionian, Adriatic, Aegean. Because the Strait of Sicily (see St. Sicily) does not belong to a particular biogeographical zone, tows sampled in this area have only been considered in the analyses conducted at basin scale.

We analysed data collected from annual bottom trawl surveys performed in spring (May-June) from 1996 to 1999 over the upper and middle slope (200 to 800 m depth) of the northern Mediterranean Sea, ranging from 36.3 to 45.7° N, and 5.3°W to 28°E, within the framework of the MEDITS project [38]. This research programme is the first scientific survey providing both high resolution and standardized data at large scale from the Mediterranean Sea (and one of the few anywhere in the world at such a large scale). The large MEDITS study zone (Fig. 1) has been divided into operative sub-areas (see [38]). In each sub-area, a stratified random-sampling design based on bathymetric strata (200–500 m and 500–800 m) was applied annually. Information recorded by an underwater Scanmar system - to control the trawl geometry (horizontal and vertical openings, contact with the bottom) - allowed us to exclude the hauls that had not been properly carried out. Analyses were performed on 1454 hauls (see Text S1 for details on hauls selection).

We have considered two spatial scales: basins and biogeographical zones (Fig. 1). Boundaries between basins and biogeographical zones correspond to those often adopted in previous works (see references in [39]). The sampling procedures of these surveys were standardised according to a common protocol including the use of the same gear and the same sampling strategy for the whole zone studied. For the continental slope, the duration of the hauls of the MEDITS surveys was standardized at 60 minutes whereas it was 30 minutes on the shelf (see [38] for full details). To date, the rare previous diversity studies conducted at the scale of the whole MEDITS area on the basis of data of similar quality were exclusively focused on the continental shelf (∼ 10 to 200 m depth, see 6 and 30), while the present work deals with the continental slope. In addition, working at the scale of the whole MEDITS area, requires the use of several vessels and several teams in order to complete the sampling of the whole zone during a short period of the year (May-June). In this context, we have restricted our analysis to a large sub-set of 76 groundfish species (see 1) properly sampled by all the teams involved in such a way as to strictly limit the risk of a variability of accuracy in sampling identification between the different teams.

### Descriptors Considered and Statistical Analyses

The first two stages of our work are based on a recently conceived approach [40] that simultaneously analyses several widely used diversity indices related to four major aspects (or components) of species diversity (species richness, rarity, evenness and species taxonomy) in order to identify a set of complementary diversity indices.

In a first stage, our approach involves a pre-selection among numerous existing indices of those that present complementary theoretical properties (and possibly complementary drawbacks). These indices were selected on the basis of (1) the expected complementarity of their conceptual and statistical properties (as described in the literature), and (2) the nature of the data available for our work (i.e. abundance per species). This stage led us to define an initial set of 11 indices (see Table 1). Of course, other diversity indices (e.g. Chao2, Hill numbers) could have been added and/or have replaced some of the indices featuring in the initial list (but see Text S1 for a brief discussion of the main properties of the indices included in the initial list, their original references and their complementarity/redundancy with other existing measures).

**Table 1:** Species diversity components and descriptors studied.

Component	Descriptor name	Formula	Expected properties	Reference
Species richness	Species density^1^	*S* = Number of species by haul	Standardize species richness per unit area	
	Margalef	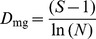	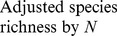	Margalef (1958)
Rarity	Rarity^1^	*rarity* = number of species with less than 5% occurrence	Define rarity in term of species range size	
Evenness	Heip	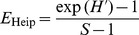	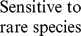	Heip (1974)
	Berger Parker	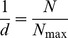	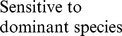	Berger and Parker (1970)
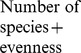	Shannon-Wiener	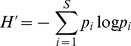	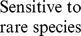	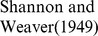
	Simpson diversity	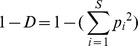	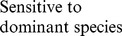	Simpson (1949)
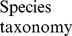	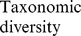	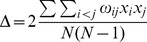	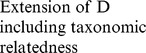	Warwick and Clarke (1995)
	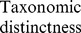	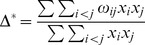	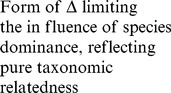	Warwick and Clarke (1995)
	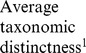	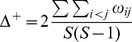	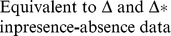	Clarke and Warwick (1998)
	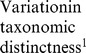	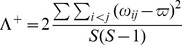 where 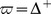	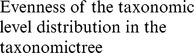	Clarke and Warwick (2001)

*x_i_* (*i* = 1, …, *S*) denotes the abundance of the *i*th species, *N* ( = 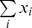) is the total number of individuals in the sample. *p_i_* ( =  *x_i/_N*) is the proportion of all individuals belonging to species *i, N*_max_ is the number of individuals of the most abundant species, *ω_ij_* is the “distinctness weight” given to the path length linking species *i* to the first common node with species *j* in the hierarchical classification. Double summations are over all pairs of species *i* and *j* (note that the distance between 2 individuals of the same species is set to 0). Indices based on presence-absence data are marked by ^1^. See detailed properties and original references of the indices in Magurran (2004) and in Mérigot *et al.* (2007b).

In a second stage, the investigation of the empirical relationships between the 11 indices included in the initial list has enabled us to select a shorter list of indices that show complementary diversity patterns on the data analyzed. It was based on the use of both a principal component analysis (PCA) based on rank correlation matrix [41], and on an in-depth analysis of the Spearman rank correlation matrix (see [40] for further information). We assumed that a Spearman rank correlation coefficient approximately <0.5 meant that an important part of the information provided by the two corresponding indices remained complementary and might justify keeping the two indices in the analysis of diversity patterns (see [6] for further details on this rationale). In contrast to the first stage, where the pre-selection was based on external knowledge (e.g. theoretical properties of the indices, nature and accuracy of the available data), the second stage is directly driven by the analysis of the data sampled in the field.

In a third stage, we have analysed the variations of each of the complementary diversity indices in function of depth, biogeographical zone, basin, and year. In the Mediterranean Sea, knowing the large-scale eastward decline in primary production (e.g. [42]), comparing the level of species diversity between areas according to the longitudinal axis is a practical and widely used basis for discussing the expected major influence of variation in primary production on the structuring of large-scale species diversity patterns. Otherwise, it is worth noting that the aim of working on 4 annual surveys is not to accurately analyse inter-annual variations of the diversity patterns. Here the “year” effect is only considered with a view to assessing whether both the correlations between diversity indices and the spatial pattern of each of the complementary groups of indices are reproducible from one survey to another (and are not induced either by random or exceptional factors). Because the scatterplots of each index versus depth showed a non-linear trend, Generalized Additive Models (GAM, [43]) were performed to analyse the bathymetric patterns. These models do not impose a parametric form on the effects of the continuous variable depth. Here, the non-linear effects of depth were fitted using “loess”, a local weighted regression method. The variables “year” and “biogeographical zone” (or « Topographical basin » depending on the scale studied) were considered as discrete factors with 4 and 5 (or 2) levels respectively. Additivity of models enables us to investigate the effect of each variable/factor after removing the effects of the two others. Interactions between variables were not kept in the models, because parameter values of interaction were very weak relatively to the individual variable effects, and thus considered as negligible.

Differences of diversity indices values between areas (i.e. between basins and between biogeographical zones) were analysed by means of a non-parametric ANOVA (Kruskal-Wallis test). The GAM were computed using the SAS statistical system, while all the other statistical analyses and diversity indices mentioned above were performed using R software (R development Core Team, 2012).

## Results

### Multicomponent Aspect of Species Diversity

**Figure 2:**
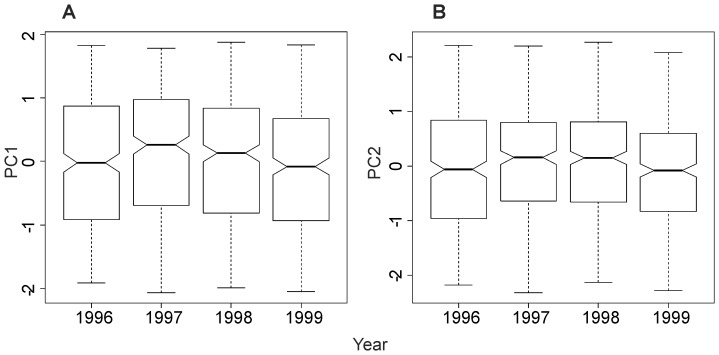
Variation of PCA scores according to years. (A) axis 1 (43%), (B) axis 2 (22%).

**Figure 3:**
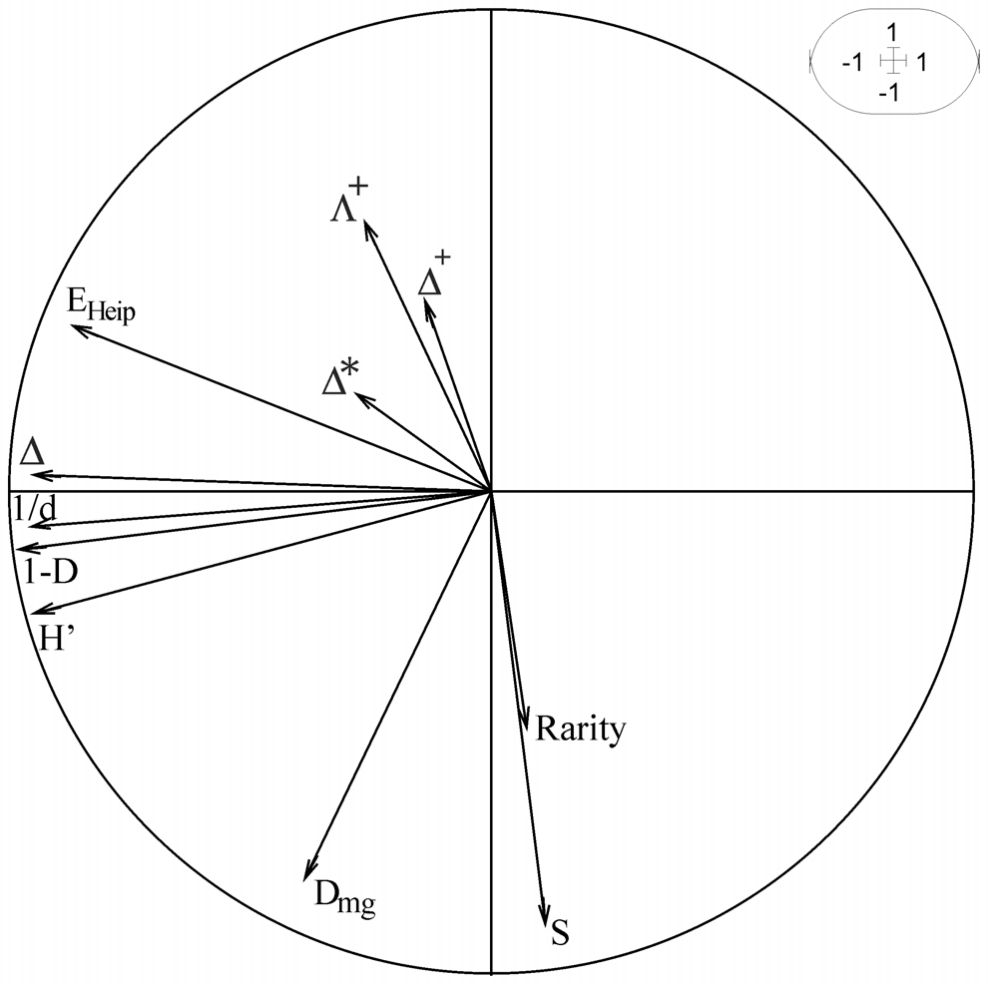
Projection of the diversity indices onto the first factorial plan of the PCA. (axis 1: horizontal –43%, axis 2: vertical –22%). Codes of diversity indices are given in Table 1.

The detailed analysis of the PCA was restricted to the first two components that accounted for 65% of the total inertia. These first two principal components were not exposed to strong temporal variability (Fig. 2), showing that the relationships observed between indices were stable during the course of the study. The first principal component (43%, Fig. 3) was strongly correlated with all evenness indices (1/*d* and *E*_Heip_), the two heterogeneous indices (*H’* and 1-*D*) and with one of the taxonomic diversity indices (Δ). The second principal component (22%) was mainly correlated with the two indices focused on the number of species (*S* and *D*_mg_) which were, in general, weakly correlated with all the other diversity indices (Table 2). This result showed that indices focused on the number of species (*S* and *D*_mg_) exhibited a different response from that given by the other diversity components. Similarly, the Spearman’s correlations matrix also showed that each of the four other indices (Δ^*^, Δ^+^, Λ^+^ and *Rarity*) provided complementary information on groundfish species diversity in the continental slope of the whole northern Mediterranean Sea (Table 2). It is worth noting that the correlations between indices might appear to be significant, even for low correlation values (e.g. between *S* and *H’*, where Spearman coefficient = 0.10 but *p*<0.05). This mainly resulted from the high number of observations analyzed (i.e. 1454 samples) that increased the power of the statistical tests. In short, the simultaneous analysis of PCA and Spearman correlation coefficients provided a basis for roughly grouping the 11 species diversity descriptors studied into 6 components that provided information on species diversity that were mainly complementary to each other: (1) number of species (*S* and *D*_mg_), (2) evenness (*E*_Heip_, 1/*d*, *H’*, 1-*D*) and Δ, (3) *rarity* and each of the three other measures of taxonomy : (4) Δ^*^, (5) Δ^+^, and (6) Λ^+^.

**Table 2:** Spearman rank correlation coefficients calculated between all the diversity indices.

	*S*	*D*_mg_	*H’*	1-*D*	*E*_Heip_	1/*d*	Δ	Δ*	Δ^+^	Λ^+^	*Rarity*
*S*	1										
*D*_mg_	0.73	1									
*H’*	0.10	0.56	1								
1-*D*	−0.03	0.42	0.97	1							
*E*_Heip_	−0.52	0.03	0.75	0.82	1						
1/*d*	−0.07	0.35	0.91	0.97	0.80	1					
Δ	−0.10	0.32	0.86	0.89	0.78	0.87	1				
Δ*	−0.05	−0.01	0.12	0.13	0.12	0.13	0.50	1			
Δ^+^	−0.17	−0.13	0	0.03	0.10	0.04	0.20	0.40	1		
Λ^+^	−0.37	−0.16	0.10	0.15	0.34	0.17	0.21	0.13	0.38	1	
*Rarity*	0.37	0.27	0.01	−0.04	−0.21	−0.04	−0.05	0.01	0.07	−0.15	1

All correlations are significantly different from zero (with *p*<0.01), except for underlined values. The Spearman coefficient distribution under null hypothesis was approximated by a normal distribution with mean equal to 0 and standard deviation equal to 1/√ (n–1). Codes of diversity indices are given in Table 1.

### Organizational Patterns

The bathymetric and geographical patterns of each of the groups of indices that provided complementary information on slope fish species diversity was investigated through the analysis of a single index : *S*, 1/*d,* Δ^*^, Δ^+^, Λ^+^. Only the rarity component was not considered hereafter. The strong variability of the index of rarity did not allow us to properly describe its spatial patterns (the standard deviations of *Rarity* in Table 3 provide an illustration of this problem).

**Table 3:** Spatial heterogeneity of the indices at the two scales studied (mean values±SD).

	Iberian-Lions	Tyrrhen	Ionian	Adriatic	Aegean	Tests Biogeographical zone scale			Western basin	Eastern basin	Tests at Basin scale			North Mediterranean Sea
	(113)	(668)	(199)	(164)	(187)	Chi-2	*p*		(781)	(450)	Chi-2	*p*		(1231)
*S _mean_*	14.59±4.18	14.15±4.21	13.16±4.47	14.02±3.81	14.25±5.80	15.16	0.0097		14.22±4.21	14±4.88	6.47	0.01		14.12±4.53
1/*d*	2.57±0.99	2.18±0.82	2.88±0.96	2.87±0.97	2.25±0.90	63.88	1.316 × 10^−15^		2.23±0.86	2.62±0.97	63.88	1.316 × 10^−15^		2.41±0.93
Δ^*^	68.17±7.44	64.72±6.12	65.75±5.46	66.33±6.07	63.44±8.34	0.07	0.78	ns	65.22±6.43	64.37±6.96	0.08	0.78	ns	64.82±6.70
Δ^+^	68.57±2.89	68.48±2.62	67.79±3.12	67.90±2.56	69.44±3.26	0.007	0.93	ns	68.49±2.66	68.47±3.06	0.01	0.93	ns	68.49±2.85
Λ^+^	127.51±46.12	131.93±53.70	137.70±63.40	135.99±57.59	132.30±48.38	0.88	0.34	ns	131.30±52.67	134.15±56.54	0.88	0.34	ns	132.62±54.50
*Rarity*	0.55±0.70	0.29±0.56	0.36±0.60	0.41±0.77	0.46±0.87	70.83	6.858× 10^−14^		0.33±0.59	0.50±0.83	11.18	0.0008		0.41±0.72

Kruskal-Wallis tests; ns = non significant effect (*p*>0.01). Number of hauls is given in brackets. *S mean* : mean number of species by haul for the corresponding spatial unit. The codes of the other diversity indices are given in Table 1.

#### Bathymetric trends

Most of the complementary indices studied showed significant variation according to depth, biogeographical zone and year (Table 4). In all cases, bathymetry was the most explicative factor while the “year” effect was the least important and could be considered as negligible (see Table 4). Among the three effects studied, the influence of depth strongly dominated for *S*, Λ^+^, and, to a lesser extent, for Δ^+^. On the other hand, the general models (i.e. including the three factors studied: bathymetry, area and year) only explained a limited part of the total variability of each of the complementary components studied, (between 7.5 and 41%) suggesting that other factors might significantly impact diversity patterns.

**Table 4:** General results for GAM models of diversity indices.

	*S*	1/*d*	Δ^+^	Δ^*^	Λ^+^	df
Null model	1922	1187	10507	56879	3841249	1330
General Model	589 (30)	218 (18.3)	1896 (18)	4280 (7.5)	1592003 (41)	11
Depth	526 (27)	121 (10)	1099(10.5)	1896 (3.3)	1492515 (38.8)	4
Biogeographicalzone	41 (2 )	93 (8)	544 (5.5)	1801 (3.1)	90907 (2)	4
Year	22 (1)	4 (0.3) ns	252 (2 )	582 (1.1)	8580 (0.2)ns	3

Deviance for Null model. ΔDeviance for the General model (including all the three variables/factors) and for each of the separated factors/variables. df: degree of freedom. ns: non significant effect when *p* (> ΔDeviance) >0.01. Percentage of the deviance of diversity indices explained by the factors/variables studied are given in brackets.

**Figure 4:**
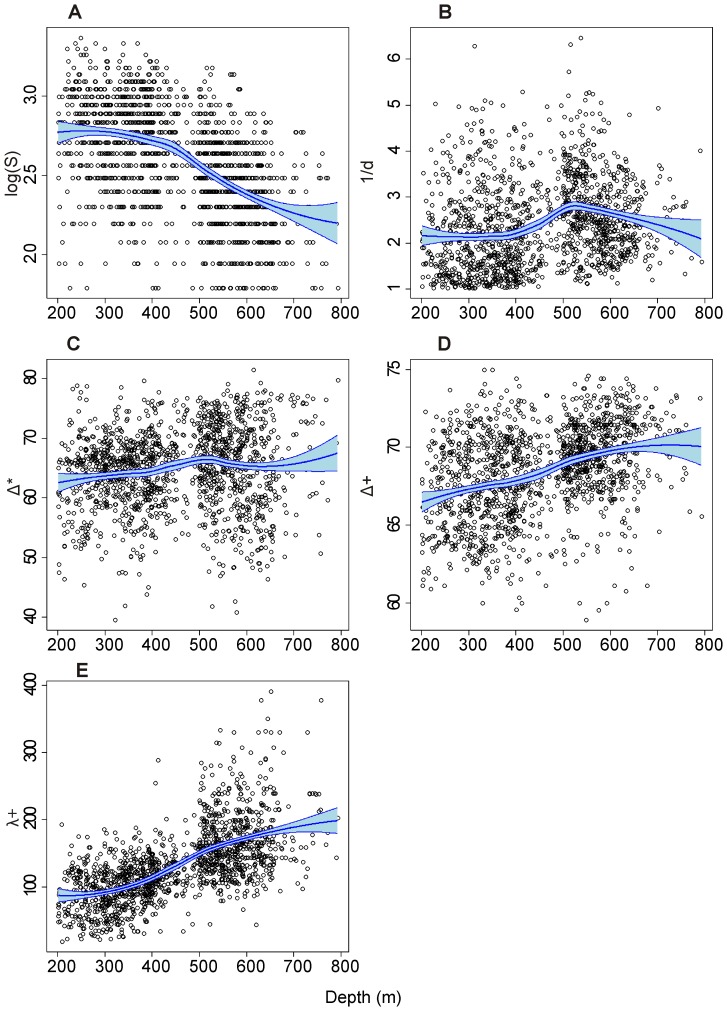
Bathymetric trends in diversity. Variation of the selected indices (ordonnate) according to depth (abscissa) without neutralisation of the effects of year and biogeographical zones (Loess curves with confidence interval). (A) log S, (B) 1/*d*, (C) Δ^*^ (D) Δ^+^, (E) Λ^+^. Codes of diversity indices are given in Table 1.

Bathymetric trends of each diversity index (Fig. 4) were very similar to those observed after removing the effects of year and biogeographical zones (using GAM, figure not provided) showing that these 2 latter factors did not strongly alter the bathymetric pattern of the diversity indices. The indices considered can be split into three categories according to their variation with bathymetry: (1) index showing a decreasing tendency with depth (i.e. *S*), (2) indices showing the opposite trend (Δ^*^, Δ^+^, and Λ^+^), and (3) index showing a clear non-linear trend (i.e. 1/*d* with a maximum around 500–550 m depth).

More specifically, on average, species richness decreased from ∼14.5 species at 200 m (i.e. exp^2.8^) to ∼ 9 species at ∼ 750 m (i.e. exp^2.2^; see Fig. 4). Otherwise, the inverse of Berger-Parker reached its maximum values at around 530 m depth (*1/d* ∼ 2.8). This meant that this depth was marked by the minimum values of dominance (*d* ∼ 0.36). On average, 36% of the individuals belong to the dominant species for depths close to 530 m, while dominance was higher for both shallow (∼ 49% between 200 and 380 m depth) and deeper waters (∼ 43% around 750 m depth).

Each of the three indices of taxonomic diversity increased with depth. However, the extent of the increase of indices values was usually weak from an ecological viewpoint. For instance, mean values of Δ^*^ increased from 63 (200 m depth) to ∼ 66 (∼770 m depth). This meant that 2 individuals belonging to two different species randomly sampled were separated on average by 3.15 for 200 m depth (i.e. 63/20), and by 3.3 (i.e. 66/20) hierarchical levels for ∼770 m depth (see Text S1 for explanation of taxonomic indices computation and meaning). A similar statement can be made for Δ^+^.

#### Longitudinal pattern

**Figure 5:**
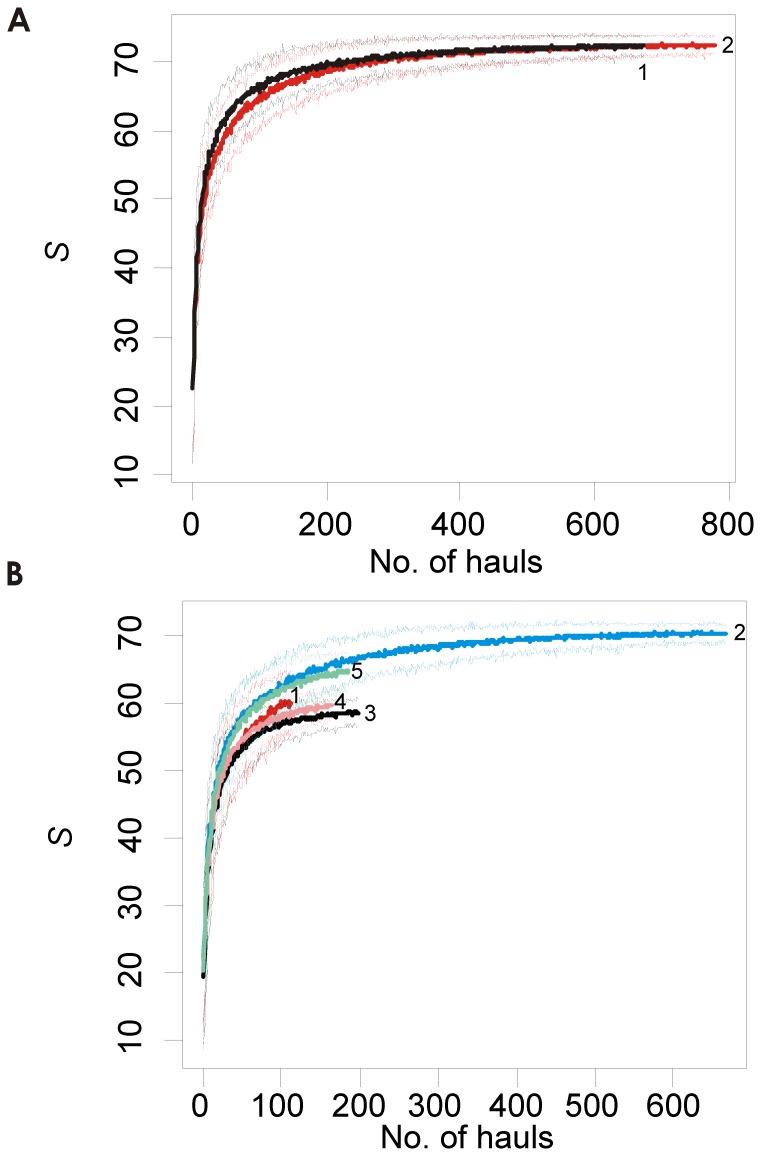
Geographical patterns in species richness. Rarefaction curves for each area at (a) basin scale (1 - western basin; 2 - eastern basin) and (b) biogeographical zone scale (1 - Iberian-Lions zone; 2 - Tyrrhenian; 3 -Ionian; 4– Adriatic; 5 - Aegean). Plotted values in ordinate are means of 50 estimates of the number of species (thick curves) and its confidence interval (thin curves), based on 50 randomizations (with replacement) of the stations sampled in each area, according to the number of hauls sampled (on the abscissa).

Our results highlighted the absence of a west-east decreasing trend for all indices analysed, at both basin and biogeographical zone scales. We investigated species richness patterns on the basis of both total species richness per area (by mean of sample-based rarefaction curves, Fig. 5) and mean species richness per surface unit (i.e. trawl hauls, Table 3). Rarefaction curves did not reach an asymptotic maximum. However, most of the curves suggested that increasing the sampling effort may reveal few additional species, showing that the areas studied have been thoroughly sampled by these surveys (for the analysis at basin scale) or at least that the sampling effort was extensive enough to provide a basis for roughly ranking the areas (for the analysis at the scale of the biogeographical zones, except for the Iberian-Lions zone). At basin scale, no difference in rarified total species richness appeared between the western and the eastern basins (Fig. 5a). At the scale of the biogeographical zone, the Adriatic and the Ionian Seas appeared to be the poorest biogeographical zones in the northern Mediterranean Sea, while the Tyrrhenian and the Aegean Sea were the richest (Fig. 5b). At both scales studied, spatial trends observed when comparing estimates of total species richness per area were roughly consistent with those observed when comparing results for mean species richness per surface unit (i.e. trawl hauls) for the different areas (see Table 3). The main exception concerned the position of the Iberian-Lions zone which showed the highest values of all the biogeographical zones when comparing the mean number of species per surface unit per area.

**Figure 6:**
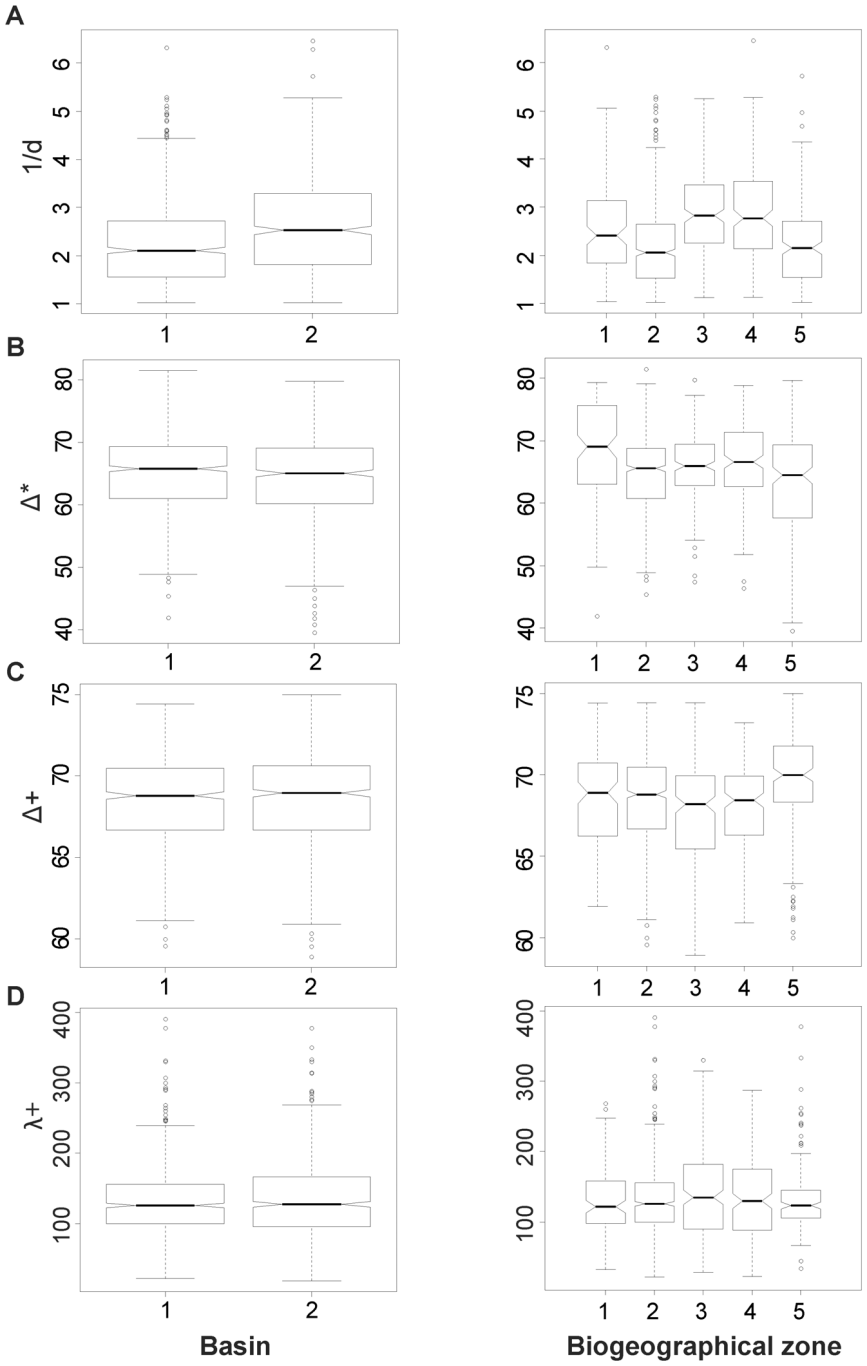
Geographical pattern in species diversity. Box-plot for (A) 1/*d*, (B) Δ^*^, (C) Δ^+^, (D) Λ^+^ at the scale of the basin (left column), and the biogeographical zones (right column). Basin and biogeographical zone codes as in Fig. 5 Codes of diversity indices are given in Table 1.

Likewise, the inverse of Berger-Parker (1/*d*) – which represented the evenness component - showed no decrease between west and east at both basin and biogeographical zone scales (Kruskal-Wallis tests, Table 3). 1/*d* was significantly higher in the eastern basin than in the western basin, mainly because of the situation in both the Adriatic and Ionian seas (Fig. 6). At basin scale, although significant, the variations in evenness/dominance were limited (Table 3): mean values (± SD) of Berger-parker (i.e. *d* and not 1/*d*) range between 0.45±0.19 (western basin) and 0.38±0.17 (eastern basin). This showed that, on average, the most dominant species represented 45% and 38% of the total number of individuals for the western and eastern basins respectively. Finally, Δ^*^, Δ^+^, and Λ^+^ showed no significant variation at either basin or biogeographical scale (Kruskal-Wallis tests, Table 3).

## Discussion

### Variability of Bathymetric Patterns of Diversity

While in numerous studies, species richness is still generally used as the only descriptor for studying bathymetric trends in species diversity, we found that the complementary diversity components studied did not show a single bathymetric pattern. This result clearly suggests that species richness cannot be used as a general proxy for investigating the full complexity of bathymetric variation in species diversity in the ecosystem studied.

According to the most popular theory, bathymetric patterns of species richness are expected to be described by hump-shaped curves, so that peak diversity occurs at some intermediate level. Thus, in his review, focusing on soft-bottom benthic invertebrates, [8] noted that the expected general pattern is that species richness changes with depth, increasing from 200 m to 1500–2500 m, and then decreasing as depth increases. In a large scale analysis of groundfishes in the oceans surrounding New-Zealand, [12] also found hump-shaped patterns but with highest richness occurring at depths of 900 to 1000 m. Hump-shaped patterns of diversity might be due to the occurrence of favourable environmental conditions, such as maximum productivity, near the middle of the depth gradient [44]. Alternatively, they might potentially be due to a mid-domain effect: the tendency for overlap to be highest near the mid-point if species ranges are distributed randomly within some spatial domain [18]. The depth range of our study (200 to 800 m) is too restricted to enable us to fully take part in this debate. However, the decreasing trend we observed for species richness in this bathymetric range is clearly in contrast to what might be expected on the basis of the hump-shaped theory. More generally, a non-exhaustive analysis of the literature devoted to demersal fish assemblages over all - or part of - the same bathymetric range as our study revealed a wide variety of relationships between the number of fish species and bathymetry on the continental slopes of the world ocean. Depending on the study, some authors have observed a positive relationship [11], [45], [46], a negative relationship [10], [30], [37], [47], a hump-shaped relationship [12] or even no relationship at all (e.g. [34]). In short, our results – supported by the strong variability of the species richness bathymetric patterns observed in the literature - clearly highlight the need to re-examine the validity - or at least the general applicability – of the “hump-shaped” diversity theory.

Concerning the other complementary diversity components analyzed, the inverse of Berger-Parker (1/*d*) slightly peaks around 530 m depth, while the other indices showed a slight increasing trend within the bathymetric range studied. In the literature, analysis of the link between bathymetry and diversity components other than species richness, while poorly described, also seemed to be context-dependent. This is notably the case for evenness (see [48], for positive correlation; [47] or [49] for negative correlation; [45] for non-monotonic relationship; [11] for no relationships in 4 of its 5 areas studied). Concerning taxonomic indices, [50] found hump-shaped patterns, with highest values of Δ^+^ and Λ^+^ occurring at depths of 500 and ∼ 300 m respectively. Here we found a different result, with a slight and progressively increasing trend for both Δ^+^ and Λ^+^ between 200 and 800 m depth.

Whatever the diversity index considered, the strong variability of the bathymetric patterns of slope groundfish species diversity observed in the world ocean, combined with our own results (i.e. usually weak bathymetric trends between ∼ 200 and 800 m for each of the complementary diversity components analyzed), leads us to the following conclusion. In contrast to its predominant role in the spatial distribution of species composition (notably described within the bathymetric range of our study), depth cannot any longer be considered as the single most important environmental predictor of variation in groundfish diversity along the upper and middle slope. Other factors, partly varying with bathymetry, but also with the specific context, such as environmental and anthropogenic activities [12], [51], may well partly explain the variability of the bathymetric pattern of demersal fish species diversity observed from one study to another.

### Longitudinal Trend in Species Diversity: New Insights for Mediterranean Fish

We did not find the extensively described longitudinal eastwards decline in species richness, which is considered as a “paradigm” of the large-scale distribution of diversity for numerous benthic groups in the Mediterranean Sea (see references in [28], [29] or [39]). In contrast, our results are in agreement with one of our previous studies, also conducted on the basis of a set of standardized and high-resolution data in the whole northern Mediterranean Sea, but focused on fishes of the continental shelf [39]. The similarity of the results we obtained for both the continental shelf and slope, strongly suggests that the general conclusion that large-scale species richness patterns in the Mediterranean Sea show a single decreasing trend eastwards might not hold true for northern Mediterranean groundfishes.

Whatever the taxa studied (fishes, invertebrates), the large-scale eastwards decreasing trend in species richness found by previous studies was primarily related to a similar decreasing west-east gradient of biological production (see for instance [28]). This widely admitted hypothesis was notably consistent with the strong difference in primary production and nutrient availability observed between eastern basin (about 150 mg C/m^2^/d, see [42]) and western basin (about 350–450 mg C/m^2^/d, see [42]). High productivity is supposed to support higher growth rates that lead to a diverse array in the dynamics of predation and competition and ultimately to comparatively high species richness [16] **.** In contrast, in our work, at both scales of observation, only Δ^*^ showed a significant – but very limited – eastwards decreasing trend at the scale of the biogeographical zone. The general lack of a west-east decreasing trend in species diversity we found does not necessarily mean that biological production does not affect diversity patterns, in as much as its impact might be masked by other factors. However, this finding emphasizes that differences in biological production (or more generally in food availability) should not be invoked as a major factor to explain large scale patterns in most of the complementary components of the fish diversity that we analyzed in the northern Mediterranean Sea.

In addition, it is worth noting that, the vast majority of the previous Mediterranean large-scale studies, including most of the recent ones, were restricted to the empirical comparison of heterogeneous pre-existing regional data sets, collected with different sampling design and for different purposes. To our knowledge, with the exception of the recent work of [9], all these studies (whatever the taxa considered) found the expected large-scale longitudinal decreasing trend in species richness (notably for fishes, see for instance [7], [22], [52]). The fact that areas in the western Mediterranean Sea have been more widely investigated by scientific surveys than the eastern areas [29], [39], [53] might strongly explain, the eastwards decreasing trend described by studies based on the compilation of previously published local data bases. More generally, this result raises the question of the suitability of a very widespread approach in ecology, based on the empirical mixing of heterogeneous data sets, to properly describe large-scale diversity patterns, in particular when dealing with indices that are very sensitive to sampling design and effort, such as species richness.

### The Multicomponent Aspect of Slope Fish Diversity: Towards a New Approach to Monitoring Survey Design

We found that the indices used in our work might be split into 6 complementary groups of descriptors of slope fish diversity. These conclusions are similar to those of recent studies using the same multi-component approach, both for demersal fishes of the continental shelf in the Mediterranean Sea at regional [36], [40] and large scales [6], and also for pelagic fishes of the open sea in the Indian Ocean [54]. We found a high level of reproducibility of the number and the nature of the complementary diversity components evidenced for these different fish communities (demersal, pelagic), scales (local, regional, large), and habitats (coastal, open-ocean and now continental slope). This result justifies continuing to work along these lines with a view to assessing to what extent (i.e. for which range of fish communities, areas and scales) it would be feasible to define a general shortlist of indices that could be used as a common basis for monitoring the multicomponent aspect of fish diversity in the future for different management scales and for different ecosystems. Establishing a shortlist of complementary indices as guidelines for investigating the multicomponent aspect of diversity for a predefined range of situations would strongly facilitate comparisons between future studies (notably for the purpose of large-scale comparisons). The list of initial indices to be used (here 11) could of course be enriched by indices that cover other facets of diversity (e.g. functional diversity, see [2]) in order to encompass as wide a range as possible of objectives and data types.

In conclusion, this work provides the first quantitative reference picture of the spatial distribution of groundfish diversity on the continental slope of the whole northern Mediterranean sea on the basis of a standardized data set. This baseline not only enriches our knowledge on slope diversity patterns through the implementation of several diversity components, but it also radically alters the general perception of large-scale species richness patterns in the Mediterranean. Our results support the need to revisit both the paradigm based on the eastwards decreasing trend in species richness through the Mediterranean Sea and the expected dominant role played by primary production with regard to large-scale diversity patterns in this sea. More generally, we have demonstrated that the components of species diversity we analyzed did not always show a consistent pattern of distribution according to either depth or to spatial area, suggesting that they are not driven by the same factors (or at least not in the same way). This finding, which implies that different complementary diversity components may respond differently to external driving forces, may have strong implications in terms of diversity monitoring and management. Firstly, it specifically shows the need to extend the number of indices traditionally considered in the monitoring of slope fish biodiversity to a broader set of indices that exhibit complementary responses in the field. Secondly, it calls for the development of lines of new research in order to identify among the complementary indices those that are the most sensitive to the potentially most important structuring factors (fishing, global change, etc.). *In fine,* such an approach could radically alter both the definition and the identification of biodiversity hotspots, and finally the practical delimitation of priority zones for protection.

## Supporting Information

Table S1 **List of the species considered.**
(DOCX)

Text S1 S**election of the set of indices considered: properties and complementarity/redundancy with others existing measures.**
(DOCX)
