# Correction: Large-Scale Diversity of Slope Fishes: Pattern Inconsistency between Multiple Diversity Indices

**DOI:** 10.1371/journal.pone.0191588

**Published:** 2018-01-17

**Authors:** Jean-Claude Gaertner, Porzia Maiorano, Bastien Mérigot, Francesco Colloca, Chrissi-Yianna Politou, Luis Gil De Sola, Jacques A. Bertrand, Matteo Murenu, Jean-Pierre Durbec, Argyris Kallianiotis, Alessandro Mannini

The second author’s name is spelled incorrectly. The correct name is: Porzia Maiorano.

There is an error in affiliation 3 for author Porzia Maiorano. Affiliation 3 should be: Department of Biology, University of Bari Aldo Moro, Bari, Italy.
