# Supplementary material for: Large-Scale Diversity of Slope Fishes: Pattern Inconsistency between Multiple Diversity Indices
Source: PLoS One. 2013 Jul 3;8(7):e66753. doi: 10.1371/journal.pone.0066753 (PMC3700978; doi:10.1371/journal.pone.0066753)
Supplement: Table S1 — List of the species considered. (DOCX) [file pone.0066753.s001.docx]

**Table S1** - **List of the species considered**

*Alepocephalus rostratus** Risso1820

*Argentina sp*

*Arnoglossus laterna* (Walbaum 1792)

*Arnoglossus rueppelii* (Cocco 1844)

*Aspitrigla cuculus* (Linnaeus 1758)

*Bathypterois dubius* Vaillant1888*

*Bellottia apoda** Giglioli1883

*Benthocometes robustus** (Goode & Bean 1886)

*Coelorinchus caelorhincus* (Risso 1810)

*Callionymus sp**

*Capros aper* (Linnaeus 1758)

*Cataetyx alleni* (Byrne 1906)*

*Centracanthus cirrus** (Rafinesque 1810)

*Centrophorus granulosus* (Bloch & Schneider 1801)*

*Chelidonichthys lucernus** (Linnaeus 1758)

*Chimaera monstrosa* Linnaeus1758

*Chlorophthalmus agassizi* Bonaparte1840

*Conger conger* (Linnaeus 1758)

*Dalatias licha* (Bonnaterre 1788)

*Dipturus oxyrinchus* (Linnaeus 1758)

*Epigonus denticulatus* Dieuzeide1950

*Epigonus telescopus* (Risso 1810)

*Etmopterus spinax* (Linnaeus 1758)

*Gadella maraldi* (Risso 1810)

*Gadiculus argenteus argenteus* (Guichenot 1850)

*Gaidropsarus sp*

*Galeus melastomus* (Rafinesque 1810)

*Gnathophis mystax** (Delaroche 1809)

*Helicolenus dactylopterus dactylopterus* (Delaroche 1809)

*Heptranchias perlo** (Bonnaterre 1788)

*Hexanchus griseus** (Bonnaterre 1788)

*Hoplostethus mediterraneus mediterraneus* Cuvier1829

*Hymenocephalus italicus* Giglioli1884

*Lepidorhombus boscii* (Risso 1810)

*Lepidorhombus whiffiagonis* (Walbaum 1792)

*Lepidopus caudatus* (Euphrasen 1788)

*Lepidotrigla cavillone** (Lacepède 1801)

*Lepidotrigla dieuzeidei** (Blanc and Hureau 1973)

*Lesueurigobius sp*

*Leucoraja circularis** (Couch 1838)

*Leucoraja melitensis** (Clark 1926)

*Leucoraja naevus** (Müller & Henle 1841)

*Lophius budegassa* (Spinola 1807)

*Lophius piscatorius* (Linnaeus 1758)

*Macroramphosus scolopax* (Linnaeus 1758)

*Merluccius merluccius* (Linnaeus 1758)

*Micromesistius poutassou* (Risso 1827)

*Molva dypterygia* (Pennant 1784)

*Molva molva** (Linnaeus 1758)

*Mora moro* (Risso 1810)

*Mullus barbatus** Linnaeus 1758

*Mullus surmuletus* Linnaeus 1758

*Mustelus mustelus** (Linnaeus 1758)

*Nettastoma melanurum* Rafinesque1810

*Nezumia sp*

*Oxynotus centrina** (Linnaeus 1758)

*Pagellus acarne* (Risso 1827)

*Pagellus bogaraveo* (Brünnich 1768)

*Peristedion cataphractum* (Linnaeus 1758)

*Phycis blennoides* (Brünnich 1768)

*Raja clavata* Linnaeus 1758

*Raja miraletus** Linnaeus1758

*Raja montagui** Fowler1910

*Raja polystigma** Regan1923

*Scorpaena elongata* (Cadenat 1943)

*Scyliorhinus canicula* (Linnaeus 1758)

*Squalus acanthias* (Linnaeus 1758)

*Squalus blainville* (Risso 1827)

*Symphurus sp*

*Synchiropus phaeton (*Günther 1861)

*Torpedo marmorata** (Risso 1810)

*Torpedo nobiliana** (Bonaparte 1835)

*Trachyrincus scabrus* (Rafinesque 1810)

*Trigla lyra* (Linnaeus 1758)

*Trisopterus minutus* (Linnaeus 1758)

*Zeus faber* (Linnaeus 1758)

*Notes:* Rare species (occurrence <5 % of the tows for the whole studied zone) are marked by *. Species potentially confused in one area have been clustered at the genus level (e.g. *Symphurus sp*).
