# Supplementary material for: Large-Scale Diversity of Slope Fishes: Pattern Inconsistency between Multiple Diversity Indices
Source: PLoS One. 2013 Jul 3;8(7):e66753. doi: 10.1371/journal.pone.0066753 (PMC3700978; doi:10.1371/journal.pone.0066753)
Supplement: Text S1 — Selection of the set of indices considered: properties and complementarity/redundancy with others existing measures. (DOCX) [file pone.0066753.s002.docx]

**Text S1** - S**election of the set of indices considered: properties and complementarity/redundancy with others existing measures.**

The question of defining and choosing methods for assessing biodiversity is still the focus of widespread and very lively debate (e.g. Chao et al. 2010; Jost, 2006; Gotelli and Colwell, 2010; Hoffmann and Hoffmann, 2008; Mérigot and Gaertner, 2011; Mouchet *et al.*, 2010 ; Tuomisto 2010; Gorelick, 2011). We briefly discuss below our reasons for selecting a set of 11 indices, mentioning their main properties. We notably highlight the main differences/similarities with some other indices that are widely used in the literature and/or that have been recently promoted by several authors, in order to explain why we did not adopt them for our work.

**Species richness component**

The number of species is still the most widely used component for describing diversity in both marine and terrestrial ecosystems. Two main methods of expressing estimates of species richness have been extensively used in the literature: the number of species per specified number of individuals (numerical species richness), and the number of species per unit area (species density, Magurran, 2004).

Because species richness is sensitive to sample size [3], we have initially selected only hauls showing low variation in the surface area trawled. Among the 1515 hauls undertaken during the period studied, only 1454 have been included in the analyses (mean ± Sd : 0.08 ± 0.004 km²). We checked that swept area variation within the 1454 selected hauls did not affect values in species richness through a Chi^2^ test of independence (p > 0.05). We analysed the mean number of species by means of two indices: i) the number of species per haul as a measure of species density *S* and ii) Margalef’s species richness index *D*_mg_ (Margalef 1958) (see Table 1). Among the various indices available to adjust the number of species according to the total number of individuals sampled in each haul, we chose Margalef’s species richness index for its ease of calculation and its widespread use (Magurran, 2004). In addition, we used sample-based rarefaction curves according to the number of hauls per area to allow comparison of total species richness between areas with different sampling effort (see [3] for detailed information on this extensively used technique).

Other approaches could have been used for estimating the number of species component, such as non-parametric estimators of true total species richness (e.g. Chao or Jacknife estimators. However, here our goal was clearly not to assess the “true” total species richness, but to compare reproducible estimations of species diversity (and notably species richness) in space in order to investigate diversity patterns. For that purpose we did not deal with all the species sampled, but we have restricted our analyses to a large sub-set of 76 species. Each of these 76 species has been properly identified and sampled by each of the scientific teams involved in the large-scale survey in such a way as to strictly limit the risk of a variability in accuracy of sampling identification between the different teams. For information, we have however computed some of the most popular non-parametric estimators (see Table A below). These estimators gave exactly - or almost exactly - the same values as those of the total observed species richness we had already used (with a maximum difference of 1 species).

Table A. Estimators of true total species richness. Total observed species richness is 76.

|  | Chao | Jacknife 1 | Jacknife 2 | Bootstrap | ACE |
| --- | --- | --- | --- | --- | --- |
| Occurrence data | 76 (Chao2) | 76 | 75 | 76.15 | - |
| Abundance data | 76 (Chao1) | 76 | 76 | 76.05 | 76 |

**Rarity component**

We considered rarity which is a component that is both extensively used in conservation strategy, and is of potential interest in ecosystem functioning (e.g. Walker *et al.*, 1999; Lyons *et al.* 2005; Ellingsen *et al.*, 2007). This concept is usually defined on the basis of the level of species local abundance or the species range size (occurrence). But whatever the approach used, definitions of rarity are necessarily arbitrary (Gaston, 1994). Because species with limited occurrence are usually the most vulnerable to environmental change (Thomas and Mallorie, 1985), the occurrence criterion has been the most widely used for conservation purposes. Thus, in our study we used the criterion of occurrence (species range size) rather than local abundance to define rarity.

The definition of a threshold (rarity cutoff) to identify which species are rare (and which are not) is a necessary but subjective stage. Here, instead of *a priori* defining the rarity cutoff in a fully subjective way (as is often the case in the literature)*,* we have defined it on the basis of the empirical distribution of frequency of species occurrence (i.e. the number of species for different percentages of occurrence). The threshold of percentage of occurrence we adopted to define the list of rare species corresponded to the drop in frequency point in this distribution (i.e. where the number of species by percentage of occurrence sharply decreases when increasing this percentage). While not perfect, this threshold definition is less subjective than definitions currently available. For instance, Gaston (1994) and more recently Ellingsen et al. (2007**)**, who noted no consensus in the literature to define a threshold, proposed another subjective threshold, called the “quartile definition” (defining as rare every species with a lower abundance or occurrence below 25%). In contrast, in our study, using the above-mentioned selection method enabled us to define *a posteriori* the percentage of occurrence used as the rarity cutoff.

In this paper, the rarity index we used corresponds to the number of rare species in each sample (haul), with rare species occurring in less than 5 % of the 1454 hauls for the whole MEDITS zone (Table 1). 26 species, including 12 Chondrichthyes, were below this threshold and thus considered as rare species in our work (see Table S1).

**Species evenness component**

Evenness is complementary to the species number component in that it considers how evenly individuals are distributed among the species, and can provide complementary assessment in both monitoring species diversity and ecosystem functioning studies (Magurran, 2004; Hillebrand *et al.*, 2008; Chiarucci *et al.*, 2011). Evenness is itself considered as a multi-component concept which led us to use complementary indices to better capture this diversity component (Smith and Wilson, 1996; Beisel *et al.*, 2003). The statistical properties of evenness indices have already been extensively studied for most of the indices available, notably regarding their sensitivity to variation of rare and dominant species, and to species richness (see for instance Smith and Wilson, 1996; Ricotta *et al.*, 2001; Beisel *et al*., 2003).

In this context, we used Heip’s evenness index *E_Heip_* (Heip, 1974) which is mainly sensitive to variation in rare species (Beisel *et al.*, 2003). In contrast to the most widely used Pielou *J’* index, (Pielou, 1966), also based on the Shannon-Wiener index *H’* (Shannon and Weaver, 1949), *E_Heip_* is less sensitive to variation in the number of species (Smith and Wilson, 1996).. Otherwise, in a complementary way to *E_Heip_*, we used the *d* Berger Parker index (Berger and Parker, 1970) which is only sensitive to variations in the most dominant species (Magurran, 2004, Chiarucci *et al.*, 2011). We computed *1/d* which increases when abundances are evenly distributed (maximum diversity) among the species and decreases with dominance.

**Composite (or heterogeneous) indices**

We computed heterogeneous indices (Peet, 1974 ; Buckland *et al.* 2005), which combine both the number of species and evenness components in a single value (Table 1). We computed both Shannon-Wiener index *H’* (Shannon and Weaver, 1949) and the Simpson diversity 1-*D* (Simpson, 1949) for which there is extensive literature concerning their statistical properties, notably with regard to their sensitivity to rare and dominant species, their sensitivity to sampling size, and the relative influence of species number and evenness (Peet, 1974 ; Lande 1996 ; Lande *et al.* 2000 ; Buckland *et al.* 2005). We used Simpson diversity (*1-D*) which increases with diversity, and which is considered not to be sensitive to sample size (Lande 1996 ; Lande *et al.* 2000). *H’* is assumed to be sensitive to the changes in abundance of relatively rare species while *D* is heavily weighted towards the dominant species and less sensitive to species richness than *H’* (Peet, 1974; Boyle *et al.*, 1990). Although *H’* and *1-D* are not focused on a single diversity component, we used these two very popular heterogeneous indices to facilitate comparisons of our results with previous studies and to compare their pattern with indices dedicated to a single diversity component, i.e. species number and evenness, in order to assess the respective influence of each of these components on the heterogeneous indices on our data set.

Of course, other indices could have been used. For instance, some authors (e.g. Jost 2006, Tuomisto, 2010) recently promoted the use of Hill’s numbers (Hill, 1973) *N_a_*, such as Hill N1 [i.e. exp(*H’*) ] rather than Shannon index (*H*’). This family of indices consists of a single mathematical formula from which various indices can be derived from an *a* parameter value (e.g. N_1_=exp(*H*’), N_2_=1/*D,* etc*.*)*.* The resulting value of these indices corresponds to the equivalent number of species that would be in the sample in the case of perfect evenness, i.e. if all individuals are equally distributed among species*.* Jost (2006) proposed calling this concept and corresponding indices ‘true diversity’, also known as “effective number of species”*.* However, the concept and indices of “true diversity” have recently been contested in the literature both with regard to the terminology, the suitability of solely taking into account and applying the concept and the related indices when dealing with species diversity (e.g. Norton, 1994; Hoffmann and Hoffmann, 2008; Gorelick, 2011). Indeed*,* by considering a single concept of an “equivalent number of species”, this family of indices limits species diversity to being viewed simply as species richness, while species richness and evenness are two fundamentally different concepts and facets of species diversity. They are widely known to have different sensitivities to structuring factors, and different implications for the studied system (eg. Ma, 2005; Wilsey *et al.* 2005; Wilsey and Stirling, 2007; Moreno and Rodriguez, 2010)*.* In contrast, the approach we have adopted in our paper enables us to differentiate between these two key components of species diversity. In addition, even authors that actively promoted the use of Hill numbers, based on Jost’s work (2006), such as Tuomisto (2010) recognized that “*other measures [than true diversity] related to diversity (such as entropies and probabilities* [ie. *H*’ and 1-*D* we used]*) continue to be useful, but they represent different phenomena and should therefore be referred to by different names*”.

Similarly, Hurlbert's *PIE* (Hurlbert, 1971) (probability of an interspecific encounter) could have been preferred to Simpson (*1-D*). Hurlbert's *PIE* measures the probability that two randomly drawn individuals represent different species. From a practical viewpoint it is calculated on the basis of 1-*D* (with an adjustment for small sample size)”. These two indices are mathematically identical at the following factor $PIE=\frac{n}{n-1}\times1-D$ , with *n* the number of individuals in the sample. Consequently, because our data set is not affected by small size samples (1^st^ quartile value is 147 individuals and median value is 332 individuals for the 1454 trawls analyzed), it is thus logical that we did not use the *PIE* index.

However, we have computed *PIE* and *Hill N1* for our data in order to assess their empirical complementarity/redundancy with Simpson (*1-D*) and Shannon (*H’*), respectively (see Tables B and C). Our results unequivocally show that *Hurlbert's PIE* provides exactly the same information as *1-D,* whether we used Pearson or Spearman correlation coefficients (see Tables B and C where r > 0.99 in both cases). The same point can be made about the use of the Shannon index and the *N1 Hill* number version.

Table B. Pairwise Pearson correlation coefficients between diversity indices

|  | Shannon *H'* | Hill N1  ( exp(*H'*)) | Simpson  (*1-D*) | Hurlbert’s  *PIE* |
| --- | --- | --- | --- | --- |
| *H'* | 1.0000 | 0.9589 | 0.9632 | 0.9600 |
| *Hill N1* | 0.9589 | 1.0000 | 0.8703 | 0.8666 |
| *1-D* | 0.9632 | 0.8703 | 1.0000 | 0.9990 |
| *PIE* | 0.9600 | 0.8666 | 0.9990 | 1.0000 |

Table C. Pairwise Spearman correlation coefficient between diversity indices

|  | Shannon *H'* | Hill N1  (exp(*H'*)) | Simpson  (*1-D)* | Hurlsbert’s  *PIE* |
| --- | --- | --- | --- | --- |
| *H'* | 1.0000 | 1.0000 | 0.9709 | 0.9621 |
| *Hill N1* | 1.0000 | 1.0000 | 0.9709 | 0.9621 |
| *1-D* | 0.9709 | 0.9709 | 1.0000 | 0.9965 |
| *PIE* | 0.9621 | 0.9621 | 0.9965 | 1.0000 |

**Taxonomic diversity component**

While the above components and indices offer a complementary view of species diversity, all of them consider species as equivalent and do not take into account differences between species in their taxonomic relatedness, evolutionary history (phylogeny) or function. We did not have at our disposal phylogeny and function for the 76 species studied but “only” their taxonomy. For both their conceptual and statistical properties, we used the popular indices introduced by Warwick and Clarke (Warwick and Clarke 1995, Clarke and Warwick 1998, 2001) which for years have been widely computed in studies dealing with taxonomy (e.g. Tolimieri and Anderson 2010, and are also increasingly applied to phylogeny (e.g. Plazzi *et al.* 2010, Modica *et al.* 2011) or even functional diversity (e.g. Somerfield *et al.* 2008) in both marine and terrestrial ecosystems.

Consequently, we computed four taxonomic indices, widespread and recognized for their complementary properties, proposed by Warwick and Clarke (1995) and Clarke and Warwick (1998, 2001) that quantify the taxonomic diversity of a faunal assemblage in terms of average distance of all pairs of individuals (or species) in a sample by tracing their distances through the Linnaean taxonomic tree. Each of these four indices has specific statistical properties that were already established in Clarke and Warwick (1998, 2001).

In short, on the basis of species abundance data, Taxonomic diversity *Δ* is the average taxonomic distance (path length) traced through the taxonomic tree between two randomly chosen individuals in the assemblage, including the individuals which belong to the same species. Taxonomic distinctness *Δ** considers only individuals belonging to different species and is less sensitive to dominant species. *Δ* can be seen as a generalization of the Simpson diversity index 1-*D* (see above) incorporating the pairwise taxonomic distance between species (Table 1 in the ms, Clarke and Warwick, 1998, Warwick and Clarke, 1995). Δ differs from Δ* in its sensitivity to species dominance (Clarke and Warwick, 1998).

Because collecting presence–absence data can be easier and less time consuming than collecting abundance data, we also investigated a third index *Δ*+ in order to study the possible “loss of information” with the previous taxonomic indices that require abundance data. Average taxonomic distinctness *Δ*+ can be defined as the average taxonomic distance between two randomly chosen species in the assemblage (Clarke and Warwick, 1998). In addition to complement *Δ*+, we applied the index of Variation in taxonomic distinctness *Λ*+ which is based on the evenness of the taxonomic level distribution in the taxonomic tree, being mathematically the variance of *Δ*+ (Clarke and Warwick, 2001).

There are two main methods to define *ω_ij_* which is the weight given to the path length linking species *i* and *j* in the taxonomic tree (Clarke and Warwick, 1999), but Rogers et al. (1999) showed that Δ^+^ calculated with and without *ω_ij_* modified to reflect the quantitative reduction in taxon richness was strongly correlated. Thus, we adopted the simplest form of *ω_ij_* with equal step-lengths between each successive taxonomic level, setting the *ω_ij_* at 100 for two species connected at the highest (taxonomically coarsest) possible level (Clarke and Warwick, 1999). In this study we have considered 6 hierarchical levels (individual, species, genus, family, class, and phylum) to build the taxonomic tree. Knowing the method adopted to define the weight given to the path length linking species *i* and *j* in the taxonomic tree, each of the 5 steps necessary to link the 6 hierarchical levels was worth 20 units (i.e. 100/5).

Finally, adopting the list of 11 selected indices in the present work was also driven by the need to draw direct comparisons with our previous works that analysed the multicomponent aspect of species diversity of fishes. Keeping constant both the list of initial indices and the statistical approach for assessing the degree of complementarity/redundancy between indices offers us a unique standardized framework for analyzing the degree of reproducibility of the empirical relationships between multiple indices for different fish communities and ecosystems. Although this topic, which is directly related to the reproducibility of the multicomponent aspect of diversity, is of major concern for both theoretical ecology and monitoring plans (see Purvis and Hector, 2000; Gaertner et al. 2010; Mouchet et al. 2010; Wilsey et al. 2010), it has been very poorly investigated. Here, the comparison made between our different studies provides an important basis for discussion of the feasibility of monitoring the complementary aspects of the diversity for a given taxon, and for different situations and different management scales, on the basis of a single shortlist of indices.

**References**

Beisel, J. N., Usseglio-Polatera, P., Bachmann, V. & Moreteau, J. C. (2003) A comparative analysis of evenness index sensitivity. *International Review of Hydrobiology,* **88,** 3-15.

Berger, W. H. & Parker, F. L. (1970) Diversity of planktonic Foraminifera in deep sea sediments. *Science,* **168**, 1345–1347.

Buckland, S. T., Magurran, A. E., Green, R. E. & Fewster, R. M. (2005) Monitoring change in biodiversity through composite indices. *Philosophical* *Transactions of the Royal Society B-Biological Sciences,* **360**, 243-254.

Boyle, T. P., Smillie, G. M., Anderson, J. C. & Beeson, D. R. (1990) A sensitivity analysis of nine diversity and seven similarity indices. *Research Journal of the Water Pollution Control Federation,* **62**,749-762.

Chao, A., Chiu, C.-H. & Jost, L. (2010) Phylogenetic diversity measures based on Hill numbers. *Philosophical Transactions of the Royal Society B: Biological Sciences,* **365**, 3599-3609.

Chiarucci, A., Bacaro, G. & Scheiner, S. M. (2011). Old and new challenges in using species

diversity for assessing biodiversity. *Philosophical Transactions of the Royal Society B: Biological Sciences,* **366**, 2426–2437

Clarke, K.R. & Warwick, R.M. (1998) A taxonomic distinctness index and its statistical properties. *Journal of Applied Ecology*, **35**, 523-531.

Clarke, K.R. & Warwick, R.M. (1999) The taxonomic distinctness measure of biodiversity : weighting of step lengths between hierarchical levels. *Marine Ecology Progress Series* **184**, 21-29.

Clarke, K.R. & Warwick, R.M. (2001) A further biodiversity index applicable to species lists: variation in taxonomic distinctness. *Marine Ecology Progress Series,* **216**, 265-278.

Ellingsen, K. E., Hewitt, J. E. & Thrush, S. F. (2007) Rare species, habitat diversity and functional redundancy in marine benthos. *Journal of Sea Research,* **58**, 291-301.

Gaertner, J-C., Mérigot, B., Rélini, G., Bertrand, J.A., Mazouni, N., et al.(2010) Reproducibility of the multi-component aspect of species diversity across different areas and scales: towards the constitution of a shortlist of complementary indices for monitoring fish diversity? Ecography **33**, 1123-1135.

Gaston, K. J. (1994). *Rarity*. Chapman and Hall, London, 220 pp.

Gaston, K.J. & Spicer, V.I. (1998) *Biodiversity: An Introduction*. Blackwell Science, Oxford, 208 pp.

Gotelli, N.J. & Colwell, R.K. (2010). Estimating species richness. pp. 39-54 in: *Biological Diversity: Frontiers In Measurement And Assessment*. A.E. Magurran and B.J. McGill (eds.). Oxford University Press, Oxford. 345 pp.

Heip, C. (1974) A new index measuring evenness. *Journal of the Marine Biological Association of the United Kingdom*, **54**, 555–557.

Hill, M. (1973) Diversity and evenness: a unifying notation and its consequences. *Ecology*, **54**, 427-432.

Hillebrand, H., Bennett, D. M. & Cadotte, M. W. (2008) Consequences of dominance: a review of evenness effects on local and regional ecosystem processes. *Ecology,* **89**,1510-1520.

Hurlbert, S. H. (1971) The nonconcept of species diversity: a critique and alternative parameters. *Ecology*, **52**, 577-586.

Gorelick, R. (2011) Commentary: Do we have a consistent terminology for species diversity? The fallacy of true diversity. *Oecologia*, **167,** 885–888.

Hoffmann, S. & Hoffmann, A. (2008) Is there a “true” diversity? *Ecological Economics,* **65**, 213-215.

Jost, L. (2006) Entropy and diversity. *Oikos,* **113**, 363-375.

Lande, R. (1996) Statistics and partitioning of species diversity, and similarity among multiple communities. *Oikos,* **76**, 5–13.

Lande, R., DeVries, P. J. & Walla, T. R. (2000) When species accumulation curves intersect: implications for ranking diversity using small samples. *Oikos*, **89**, 601–605.

Lyons, K. G., Brigham, C. A., Traut, B. H. & Schwartz, M. W. (2005) Rare species and ecosystem functioning. *Conservation Biology,* **19,** 1019-1024.

Ma, M. (2005) Species richness vs evenness: independent relationship and different responses to edaphic factors. *Oikos,* **111,** 192-198.

Magurran, A.E. (2004) *Measuring biological diversity*. Blackwell Science.

Margalef, R. (1958) Information theory in ecology. *General Systems,* **3**, 36–71.

Mérigot, B. & Gaertner, J.-C. (2011) Incorporation of phylogeny in biological diversity measurement: Drawbacks of extensively used indices, and advantages of quadratic entropy. *Bioessays,* **33**, 819-822.

Modica, M. V., Bouchet, P., Cruaud, C., Utge, J. & Oliverio, M. (2011) Molecular phylogeny of the nutmeg shells (Neogastropoda, Cancellariidae). *Molecular Phylogenetics and Evolution,* **59,** 685-697.

Moreno, C. & Rodríguez, P. (2010) A consistent terminology for quantifying species diversity? *Oecologia*, **163**,279-282.

Mouchet, M., Villéger, S., Mason, M. & Mouillot, D. (2010) Functional diversity measures: an overview of their redundancy and their ability to discriminate community assembly rules. *Functional Ecology,* **24**, 867-876.

Norton, B.G. (1994) On what we should save: the role of cultures in determining conservation targets. In: Forey P, Humphries CJ, Vane-Wright RI (eds) *Systematics and conservation evaluation*. Systematics Association/Clarendon Press, Oxford, pp 23–40

Simpson, E. H. (1949) Measurement of diversity. *Nature,* **163**, 688.

Peet, R. K. (1974) The measurement of species diversity. *Annual Review Of Ecology And Systematics,* **5**, 285-307.

Pielou, E. C. (1966) Species-diversity and pattern-diversity in the study of ecological succession. *Journal of Theoretical Biology,* **10**, 370–383.

Plazzi, F., Ferrucci, R., & Passamonti, M. (2010) Phylogenetic representativeness: a new method for evaluating taxon sampling in evolutionary studies. *BMC Bioinformatics,* **11**:209.

Purvis A., Hector A (2000) Getting the measure of biodiversity. *Nature,* **405**, 212-219.

Ricotta, C., De Zuliani, E., Pacini, A. & Avena, G. C. (2001) On the mutual relatedness of evenness measures. *Community Ecology*, **2**, 51-56.

Rogers, S.I., Clarke, K.R., Reynolds, J.D., (1999) The taxonomic distinctness of coastal bottom-dwelling fish communities of the North-east Atlantic. Journal of Animal Ecology **68**, 769-782.

Shannon, C. E. & Weaver, W. (1949) The Mathematical Theory of Communication. University of Illinois, Urbana, Illinois.

Smith, B. & Wilson, J. B. (1996) A consumer's guide to evenness indices. *Oikos,* **76**,70-82.

Somerfield, P. J., Clarke, K. R., Warwick, R. M. & Dulvy, N. K. (2008) Average functional distinctness as a measure of the composition of assemblages. *Ices Journal of Marine Science,* **65**, 1462-1468.

Thomas, C.D. & Mallorie, H.C. (1985). Rarity, species richness and conservation: but-terflies of the Atlas Mountains in Morocco. *Biological Conservation* **33**, 95-117.

Tolimieri, N. & Anderson, M.J. (2010) Taxonomic Distinctness of Demersal Fishes of the California Current: Moving Beyond Simple Measures of Diversity for Marine Ecosystem-Based Management. *PLoS ONE* **5**(5): e10653. doi:10.1371/journal.pone.0010653

Tuomisto, H. (2010) A consistent terminology for quantifying species diversity? Yes, it does exist. *Oecologia,* **164**,853-860.

Walker, B., Kinzig A. & Langridge, J. (1999) Plant attribute diversity, resilience, and ecosystem function: The nature and significance of dominant and minor species. *Ecosystems,* **2**, 95-113.

Warwick, RM & Clarke, K.R. (1995) New ‘biodiversity’ measures reveal a decrease in taxonomic distinctness with increasing stress. *Marine Ecology Progress Series*, **129**, 301–305.

Wilsey, B. & Stirling, G. (2007). Species richness and evenness respond in a different manner to propagule density in developing prairie microcosm communities. *Plant Ecology*, **190**, 259-273.

Wilsey, B.J., Chalcraft, D.R., Bowles, C.M. & Willig, M.R. (2005) Relationships among indices suggest that richness is an incomplete surrogate for grassland biodiversity. *Ecology*, **86**,1178-1184.
